# Stem cell therapies: a new era in the treatment of multiple sclerosis

**DOI:** 10.3389/fneur.2024.1389697

**Published:** 2024-05-09

**Authors:** Lei Wu, Jing Lu, Tianye Lan, Dongmei Zhang, Hanying Xu, Zezheng Kang, Fang Peng, Jian Wang

**Affiliations:** ^1^Changchun University of Chinese Medicine, Changchun, China; ^2^The Affiliated Hospital to Changchun University of Traditional Chinese Medicine, Changchun, China; ^3^Hunan Provincial People's Hospital, Changsha, China

**Keywords:** multiple sclerosis, stem cell therapy, stem cell transplantation, neural stem cells, induced pluripotent stem cells

## Abstract

Multiple Sclerosis (MS) is an immune-mediated condition that persistently harms the central nervous system. While existing treatments can slow its course, a cure remains elusive. Stem cell therapy has gained attention as a promising approach, offering new perspectives with its regenerative and immunomodulatory properties. This article reviews the application of stem cells in MS, encompassing various stem cell types, therapeutic potential mechanisms, preclinical explorations, clinical research advancements, safety profiles of clinical applications, as well as limitations and challenges, aiming to provide new insights into the treatment research for MS.

## 1 Background

Multiple Sclerosis (MS), a complex autoimmune pathology, impairs the central nervous system through inflammation, demyelination, and neuronal degradation (1). Despite advancements in our comprehension of MS, formidable challenges persist in curtailing its progression and facilitating neurorestoration. Contemporary therapeutic modalities predominantly aim at symptom mitigation and disease progression containment. However, the imperative for enhanced rehabilitation and neurorestoration solutions propels the quest for innovative, efficacious therapies (2).

In recent advancements, stem cell therapy has been recognized as a frontier with significant potential in the treatment of MS. Stem cells, characterized by their inherent ability for self-renewal and pluripotency, hold promise for regenerating damaged neural tissue, modulating immune responses, and fostering an environment conducive to endogenous repair mechanisms (3). Distinct from traditional therapeutic modalities, stem cell therapy entails the transplantation of stem cells capable of differentiating into diverse neural cell types, thereby facilitating tissue regeneration (4). Moreover, these cells secrete neurotrophic factors that enhance the survival and function of adjacent neural tissue. Critically, stem cells exhibit immunomodulatory effects that attenuate inflammatory processes, offering a novel approach to mitigating the progression of MS lesions (5). Empirical studies, including laboratory and animal model research, have demonstrated the therapeutic efficacy of hematopoietic, neural, and embryonic stem cells, indicating substantial therapeutic promise (6, 7). Preliminary clinical trials have corroborated these findings, signaling a promising horizon for individuals afflicted with MS (8).

Although some reviews on stem cell therapy for MS exist, many lack comprehensiveness, depth, and focus on emerging therapeutic approaches. Existing reviews often concentrate on specific types of stem cells or particular MS subtypes, with limited integration and comparative analysis across different stem cell types. Additionally, there is insufficient attention given to a comprehensive assessment of preclinical and clinical research progress and in-depth discussions on the safety and limitations of current treatments (9, 10). Therefore, this review aims to address these gaps by offering a more comprehensive and in-depth analysis.

This review systematically evaluates the potential value and application prospects of stem cell therapy in MS management. Initially, we outline the current research progress on MS, followed by an in-depth examination of the fundamental biological characteristics, sources, and associated therapeutic mechanisms of hematopoietic, mesenchymal, neural, embryonic, and induced pluripotent stem cells. We provide a detailed analysis of the latest developments in preclinical and clinical studies, emphasizing the efficacy and safety of different stem cell types in MS treatment. Furthermore, this review identifies the primary challenges and limitations faced by current treatment methods, including ethical considerations, immune rejection reactions, and regulation of neural induction differentiation. Finally, addressing these issues, we propose directions and strategic recommendations for future research, highlighting the crucial role of innovative strategies in fully harnessing the potential of stem cell therapies. This review aims to provide profound insights and guidance for the further development of stem cell-based treatments in the MS therapeutic field, promoting their broader application and integration.

## 2 Multiple sclerosis and current landscape of treatment

The etiology of MS remains partially understood, with prevailing consensus attributing its onset to an interplay of genetic predispositions and environmental triggers (11). The hallmark pathologies of MS encompass inflammation, demyelination, and neuronal injury. Specifically, inflammation leads to lesion formation in neural tissues; demyelination results from autoimmune assaults on the myelin sheath of nerve fibers; and neuronal damage arises either directly from prolonged inflammatory states or through secondary pathological processes (12). Clinically, MS manifests in a spectrum of symptoms, including sensory deficits, motor coordination impairment, visual disturbances, fatigue, and cognitive impairments (13). The National MS Society and the MS International Federation recognize four distinct MS subtypes: clinically isolated syndrome (CIS), relapsing–remitting MS (RRMS), primary progressive MS (PPMS), and secondary progressive MS (SPMS) (14). Given the heterogeneity in symptomatology and disease trajectory, MS exerts a profound impact on patients’ quality of life, underscoring the necessity for tailored therapeutic approaches.

The current therapeutic approaches for MS include pharmacological interventions, physical therapy, and rehabilitative measures, all directed toward mitigating disease activity, relieving symptomatic burdens, and improving overall patient well-being. Among these, Disease Modifying Therapies (DMTs) are pivotal, primarily functioning to modulate the immune response and curtail inflammatory processes, thus impeding the disease progression (15). DMTs currently encompass a variety of administration routes, including subcutaneous and intramuscular injections, oral formulations, and intravenous infusions, tailored to accommodate the diverse preferences and clinical requirements of individual patients. Despite the advances in DMTs, their application is tempered by challenges related to sustained efficacy, patient-specific response variability, safety profiles, and financial implications (16). Conventional pharmacotherapy encompasses immunomodulators, anti-inflammatory compounds, and immunosuppressive medications, aimed at orchestrating immune system activity to reduce MS progression and ameliorate symptoms. An exemplar of this approach is interferon-β, which emulates endogenous interferons to temper immune hyperactivity, thereby decelerating the disease’s trajectory (17). On the other hand, immunomodulatory agents such as acetate salts function by modulating immune activity, primarily through the suppression of T-cell functionality. Despite their efficacy, there is a risk of patients developing medication resistance over time (18). During acute flare-ups, anti-inflammatory medications like methylprednisolone, a type of glucocorticoid, are utilized to mitigate inflammation; however, their long-term application is associated with adverse effects, including diminished bone density, compromised immune function, and gastrointestinal complications (19).

Beyond pharmacological interventions, holistic treatment regimens, encompassing physical therapy, rehabilitation services, and acupuncture, are employed to improve the quality of individuals with MS (20). While the efficacy of these approaches may vary across patients, the goal of achieving substantial neural regeneration remains elusive. Collectively, current therapeutic strategies can provide symptomatic relief to some degree, yet they are encumbered by various limitations and challenges. Consequently, the exploration of innovative treatments, such as stem cell therapy, represents a promising frontier in the quest for more effective MS management solutions.

## 3 Foundations of stem cell therapies

Over recent decades, the landscape of MS treatment has undergone transformative advances, with the development of a diverse array of therapeutic agents that have significantly mitigated symptoms and decelerated disease progression (2). The continuous innovation and introduction of novel pharmacological treatments highlight the evolving and dynamic nature of MS research. Despite these notable strides, the quest for effective neuroprotective and neuroregenerative interventions remains pressing. In this context, the potential of stem cell therapies has increasingly gained prominence. With their inherent capacity for pluripotency and differentiation into a myriad of cell types, stem cells stand at the vanguard of neuroregenerative medicine (21), offering unprecedented prospects for addressing the complex challenges of MS.

### 3.1 Types of stem cells

Various stem cell categories, each with unique properties, contribute differently to therapeutic applications, underscoring the diversity and versatility of stem cell-based interventions in the clinical landscape.

#### 3.1.1 Hematopoietic stem cells (HSCs)

HSCs primarily reside in the bone marrow and possess the capability to differentiate into various blood cell types, including erythrocytes, leukocytes, and platelets. These cells play a pivotal role in maintaining the homeostasis of the hematopoietic system (22). In clinical practice, hematopoietic stem cell transplantation (HSCT) has been proficiently utilized to restore or repair damaged immune systems, thereby restoring normal immune function (23). HSCT can be divided into two types based on the source of the donor cells: autologous hematopoietic stem cell transplantation (aHSCT), where the patient’s own cells are used, and allogeneic hematopoietic stem cell transplantation (allo-HSCT), involving cells from a donor. Due to its comparatively lower mortality risk, aHSCT is the preferred method for treating immune-mediated diseases (24). These interventions have demonstrated efficacy in the treatment of various hematological disorders, including leukemia and myelopathy (23). Despite initial attempts to directly employ bone marrow-derived hematopoietic stem cells for the treatment of neurological disorders, outcomes have been less than satisfactory. This is primarily attributed to the limited neurogenic potential of HSCs, rendering their differentiation into neuronal and glial lineages complex (25). As in Parkinson’s disease, although some studies have hinted at the neuroregenerative potential of HSCs, obstacles such as restricted neurogenic differentiation capacity, impermeability of the blood–brain barrier, and immune rejection challenges have hindered their widespread clinical adoption (26, 27). In recent years, there has been a renewed focus on the immunomodulatory role of HCST in the treatment of neurological disorders. By transplanting healthy HSCs to modulate the activity of the immune system, attenuate inflammatory responses, and thereby shield damaged neural tissues from immune-mediated injury (28). This novel finding expands the applicability of HSCs in neurology, providing new avenues and methodologies for the treatment of neurological disorders.

#### 3.1.2 Mesenchymal stem cells (MSCs)

MSCs are ubiquitously present in diverse biological tissues such as bone marrow, adipose tissue, and the placenta, They possess the potential for self-renewal and differentiation into mesodermal cells, capable of differentiating into various cell types including osteoblasts, adipocytes, and chondrocytes (29). Based on their source, MSCs can be classified into bone marrow-derived mesenchymal stem cells (BM-MSCs), umbilical cord-derived mesenchymal stem cells (UC-MSCs), adipose tissue-derived mesenchymal stem cells (AD-MSCs) (30), dental and oral-derived mesenchymal stem cells (31), peripheral blood-derived mesenchymal stem cells (32), muscle-derived mesenchymal stem cells (33), and lung-derived mesenchymal stem cells (34), etc. Research on these cell lineages indicates that MSCs precursors predominantly originate from perivascular cells, located in the perivascular niche, underscoring their extensive regenerative potential in adult tissues. This positions MSCs as promising therapeutic candidates for various clinical conditions (35). Notably, BM-MSCs are among the most extensively studied stem cells. Research has demonstrated that BM-MSCs serve as an effective cellular therapy for treating central nervous system (CNS) inflammation and neurodegenerative diseases (36, 37). BM-MSCs possess anti-inflammatory properties, promote the differentiation of neural stem cells, and stimulate regeneration in damaged areas of the CNS. These beneficial effects are likely mediated through paracrine signaling mechanisms and targeted migration to the injured neural tissue (37). It is worth mentioning that dental and oral-derived MSCs, such as dental pulp stem cells (DPSCs), stem cells from human exfoliated deciduous teeth (SHED), stem cells from apical papilla (SCAP), periodontal ligament stem cells (PDLSCs), gingival mesenchymal stem cells (GMSCs), and dental follicle stem cells (DFSCs), due to their embryonic neural crest origin, exhibit significant neuroregenerative potential. Therefore, they are emerging as promising cell-based therapeutic approaches for treating brain, spinal cord, cerebrovascular, and neurodegenerative diseases (31, 38–40). Despite the advantages of MSCs, their lower efficiency in differentiating into specific neural cell types limits their application in neural repair. Researchers are endeavoring to enhance the efficiency of MSC differentiation into neural cells by optimizing culture conditions and controlling differentiation pathways, aiming to further differentiate them into functional neurons for the treatment of brain and spinal cord injuries and defects (41).

#### 3.1.3 Neural stem cells (NSCs)

NSCs exhibit pluripotency with the ability to differentiate into neurons, astrocytes, and oligodendrocytes, which positions them as an optimal cellular substrate for neurologically oriented therapies (42). NSCs are naturally concentrated in specific neurogenic niches: the subventricular zone (SVZ) adjacent to the lateral ventricles and the subgranular zone (SGZ) within the hippocampal dentate gyrus (43). The therapeutic premise of NSCs utilization hinges on their capacity for neurotrophic factor secretion and differentiation into functional neural and glial cells, thereby enabling neurogenesis and the restoration of damaged CNS territories (44). NSCs have demonstrated a propensity to migrate to inflamed demyelinated regions of the CNS and differentiate into oligodendrocytes, further underscoring their therapeutic potential (45). However, the limited endogenous reservoir of NSCs available for autologous repair remains a critical limiting factor (46). Currently, research is exploring allogeneic NSC transplantation strategies, typically derived from the human fetal central nervous system (brain and/or spinal cord). Although these tissues are harvested from selectively terminated pregnancies, ethical and religious considerations pose challenges to their accessibility and ethical utilization (47). Recent studies have reported that primary NSCs can also be isolated from the cerebrospinal fluid of infants diagnosed with severe intraventricular hemorrhage (IVH) or neural tube defects (NTD) (48, 49). Given that these samples are typically discarded, the isolation of NSCs from them does not raise specific ethical concerns. Additionally, NSCs can be isolated from adult and fetal central nervous system biopsy or autopsy specimens. Studies have successfully isolated NSCs from various brain regions, including the cortex, SVZ, hippocampus, midbrain, and spinal cord (50, 51). Meanwhile, advancements in pluripotent stem cell technology are unveiling pathways to derive NSC-like progenitor cells from embryonic and induced pluripotent stem cells, thereby expanding the potential applications for neural interventions (52).

#### 3.1.4 Embryonic stem cells (ESCs)

ESCs, a class of pluripotent cells sourced from the inner cell mass (ICM) of blastocyst-stage embryos, are characterized by their indefinite self-renewal capacity and the ability to differentiate into diverse cell types, including neurons, cardiomyocytes, and hepatocytes (53). In 1981, Evans et al. first isolated mouse embryonic stem cells (mESCs) from the ICM of mouse blastocysts (54). Building on the progress in mESC research, the focus shifted to human embryonic stem cells (hESCs). Thomson et al. successfully isolated and cultured hESCs from the ICM of human blastocysts *in vitro* (55). Both mouse and human ESCs can maintain an undifferentiated state under culture conditions and differentiate into cells of all three germ layers, exhibiting self-renewal and pluripotency (54, 55). However, mESCs and hESCs exhibit significant differences in morphology, transcription, and epigenetics (56). For instance, while mESCs tend to form dome-shaped structures, hESCs typically exhibit a flat morphology. Although both express the pluripotency-associated OCT4 transcription factor, OCT4 expression in mESCs is regulated by its distal enhancers, whereas in hESCs, it is primarily regulated by its proximal enhancers (57, 58). Currently, researchers utilize a mixed culture system of LN-521 and E-cadherin to derive new hESC lines from individual blastocysts without embryo destruction. Under these culture conditions, the division rate of hESCs reaches 1:30, significantly higher than the 1:3 ratio seen with traditional methods, implying that a large number of hESCs can be obtained through fewer passages (59). With advancements in cell derivation and expansion techniques, the establishment of clinical-grade hESC banks has become feasible. A cell bank containing approximately 150 donor-derived cell lines would be sufficient to meet the therapeutic needs of most populations (60). Due to their pluripotency and compatibility with human genetics, hESCs hold vast potential for disease treatment. Particularly in the field of neurology, hESCs demonstrate significant promise. They can be directed to differentiate into neuronal cells *in vitro* (61) and integrate into neural tissues post-transplantation, promoting spinal cord proteomic repair and facilitating the recovery process (62). Their applications in neurological diseases, such as multiple sclerosis, are increasingly gaining attention, leveraging their differentiation capabilities and neuroprotective properties to counteract disease progression (63, 64).

#### 3.1.5 Induced pluripotent stem cells (iPSCs)

iPSCs are engineered from somatic cells to acquire pluripotency akin to embryonic stem cells, a breakthrough pioneered by Shinya Yamanaka in 2006. Through the introduction of a quartet of transcription factors (Oct4, Sox2, Klf4, c-Myc), mature cells such as fibroblasts or hematopoietic cells are reprogrammed into a pluripotent state (65). iPSCs’ broad differentiation capacity enables them to generate any cell type, presenting expansive utility in regenerative therapies and disease modeling (66). iPSCs originate from adult somatic cells. Various mature cell types from the human body, including umbilical cord blood cells, bone marrow cells, peripheral blood cells, fibroblasts, keratinocytes, and even cells from urine samples, can be reprogrammed into iPSCs (67–69). Specifically, urine samples offer an inexhaustible autologous cell source and demonstrate robust reprogramming capabilities. Given that iPSCs can be derived from an individual patient, they hold promise for circumventing immune rejection responses (68). The preparation process of iPSCs mainly involves somatic cell collection, gene transduction, cell culture, and differentiation induction. Firstly, samples are collected from the patient’s somatic cells (e.g., skin cells), and specific transcription factors are introduced to reprogram these cells into iPSCs. Subsequently, iPSCs can be expanded *in vitro* to form cell colonies. Lastly, scientists can guide their differentiation into the desired cell types through specific induction conditions, suitable for applications in particular therapeutic areas (70). During this process, various approaches can be employed to induce pluripotency in iPSCs, including genomic modifications to induce pluripotency, utilizing small molecules and genetic signaling pathways to promote iPSCs pluripotency, microRNAs for inducing and enhancing cell reprogramming, as well as employing chemical agents to induce and enhance the pluripotency of iPSCs (71). Consequently, unlike ESCs, iPSCs derivation circumvents ethical controversies by eschewing embryonic material, thereby offering a more acceptable alternative (72). Within neurology, iPSCs exhibit promising neuroprotective and regenerative capabilities. Neuroepithelial-like stem cells (NESCs) derived from iPSCs could stimulate damaged tissue repair and host oligodendrocyte precursor cells migration and proliferation, reduce active inflammatory cells, and promote axonal regeneration (73). Despite the burgeoning potential of iPSCs, their clinical translation is tempered by ongoing technical, safety, and efficacy evaluations, yet their research continues to pave avenues for novel neurological therapeutics.

### 3.2 Therapeutic potential of stem cells

#### 3.2.1 Differentiation

Stem cells, with their inherent self-renewal and pluripotent differentiation capabilities, emerge as a potent therapeutic modality for MS. A pivotal attribute of stem cells is their ability to morph into neurons, astrocytes, and oligodendrocytes, thereby presenting a viable strategy for neural tissue restoration and cellular repair (42). Utilizing small molecules to direct the differentiation of human iPSCs and assessing cell functionality through quantification of neural-specific markers has demonstrated successful differentiation into functional spinal cord neurons (74). Furthermore, NSCs isolated from the SGZ express MAP2, Nestin, and Pax6, exhibiting self-renewal capacity and pluripotency. These NSCs proliferate into multipolar astrocytes and neuron-like cells one week post-differentiation, and differentiate into various types of neurocytes by day 10. Notably, the majority of NSCs differentiate into astrocytes and neurons, while a smaller fraction differentiate into oligodendrocytes, with their projections intertwining to form a neural network (42). The neural differentiation potential of stem cells offers promising prospects for improving neurological disorders.

#### 3.2.2 Secretion

Stem cells demonstrate neuroprotective efficacy through the secretion of growth factors and bioactive molecules, which contribute to the mitigation of inflammatory responses, reduction of oxidative stress in neuronal cells, and establishment of a conducive milieu for neural tissue preservation (75). Research has revealed that NSCs can secrete neurotrophic factors such as nerve growth factor, brain-derived neurotrophic factor, and glial cell line-derived neurotrophic factor. These factors support axonal growth and angiogenesis in injured spinal cords, thereby promoting the repair of spinal cord injuries (44). Notably, extracellular vesicles (EVs) serve as important mediators of stem cell secretion. EVs deliver various bioactive factors via paracrine mechanisms, playing crucial roles in tissue regeneration by regulating processes such as apoptosis, inflammation, proliferation, and angiogenesis across various tissues (76, 77). MSCs are currently the primary focus of stem cell secretome-EVs. MSC-derived EVs have been shown to counteract neuronal damage and synaptic dysfunction (78). Given this promising evidence, utilizing stem cell secretome-based therapy for neurological disorders represents an innovative strategy.

#### 3.2.3 Immunomodulation

The immunomodulatory properties of stem cells are one of the key factors that make them attractive tools for cell therapy. Stem cells can exert their immunomodulatory function through mechanisms such as releasing anti-inflammatory factors and various immunoregulatory factors, and modulating the activity and quantity of immune cells (79). Studies have shown that extracellular vesicles derived from BM-MSCs lead to a significant increase in M2-related cytokines such as IL-10 and TGF-β levels, while levels of M1-related TNF-α and IL-12 are significantly reduced. This modulation polarizes microglia to alleviate inflammation and demyelination in the central nervous system of experimental autoimmune encephalomyelitis (EAE) rat models (80). Furthermore, Luz-Crawford et al. demonstrated that injection of MSCs in the EAE model promotes the generation of an immunosuppressive environment by inhibiting pro-inflammatory T cells and inducing CD4(+) CD25(+) Foxp3(+) regulatory T cells (81). These findings corroborate the therapeutic effects of stem cells on disease progression, which are associated with their immunomodulatory function.

**Figure 1:**
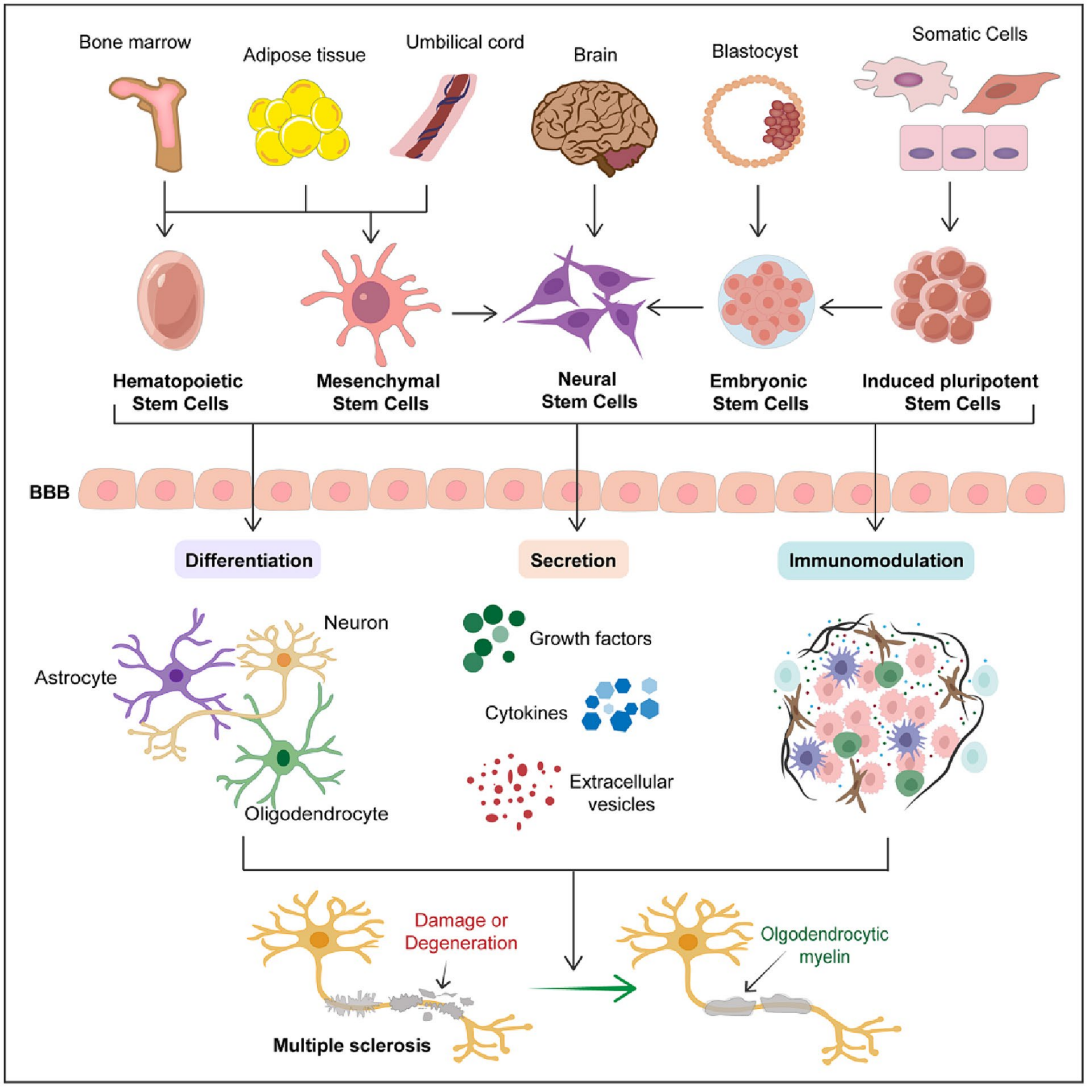
Therapeutic potential of stem cells in multiple sclerosis. The schematic diagram succinctly outlines the therapeutic potential of stem cells in multiple sclerosis. It encompasses the primary sources of various types of stem cells and the three major mechanisms through which stem cells exert their therapeutic effects in MS treatment, including differentiation into various neural cells, secretion of trophic factors, and immunomodulation. This schematic emphasizes that stem cell therapy represents a promising therapeutic strategy for the treatment of MS.

The therapeutic potential of stem cells is not solely based on single actions but often involves multifaceted therapeutic effects. By combining mechanisms such as neuro-differentiation, secretion, and immunomodulation, stem cell therapy offers possibilities for comprehensive treatment approaches for multiple sclerosis (Figure 1). Despite ongoing challenges and necessary advancements, the innovative nature of stem cell therapy makes it a promising candidate for future treatments of multiple sclerosis.

## 4 Application of stem cells in the treatment of MS

Although numerous medications exist for managing MS, they predominantly focus on halting disease progression and ameliorating symptoms, yet fail to offer a curative solution (2). The hurdles of achieving remyelination and neuronal regeneration remain significant in the MS treatment paradigm. Stem cell therapy, however, has recently surfaced as a promising innovative approach, attracting considerable interest within the medical community.

### 4.1 Application of hematopoietic stem cells in MS

In the last two decades, HSCs has gained prominence as a leading immunomodulatory therapy for autoimmune conditions (82). Specifically, aHSCT combined with immune ablation therapy has been rigorously explored for treating aggressive and treatment-resistant MS (83). There have been reports of significant symptomatic improvements in autoimmune diseases among patients undergoing HSCT for hematological cancers. This suggests the feasibility of HSCT in MS, which may offer clinical benefits (84). However, these results require further research to confirm the exact efficacy of HSCT in the treatment of MS, thus providing new directions for MS therapy.

#### 4.1.1 Procedure for HSCT

HSCT involves a multifaceted procedure that includes the mobilization and collection of HSCs, preparation of the patient through ablative conditioning, and the subsequent transplantation of stem cells (85). After cell collection, high-dose immunosuppressive therapy (HDIT) is employed to reduce the immune system activity in patients, thus creating a more favorable environment for HSCT. HDIT involves the use of high doses of immunosuppressive agents such as cyclophosphamide (CY), methotrexate, and antibodies to suppress the activity of the immune system and lower the incidence of autoimmune diseases (86). It is noteworthy that during HDIT, depletion of the immune system may trigger the expansion of myeloid-derived suppressor cells (MDSCs) which are a heterogeneous cell population including immature myeloid cells and the progenitor cells of macrophages, dendritic cells (DCs), monocytes, and neutrophils. These MDSCs can inhibit T-cell activity, modulate immune responses, and reduce inflammation (87). Yin, et al. found that accumulation of MDSCs might contribute to patients’ overall immune suppression and result in long-term survival without influence on the risk of recurrence after allo-HSCT (88). Therefore, the expansion of MDSCs during HDIT may create a more favorable environment for HSCT, thereby enhancing its efficacy. However, due to the severe toxicity associated with whole-body irradiation, HDIT has been phased out. Contemporary conditioning regimens largely rely on BEAM (carmustine, etoposide, cytarabine, melphalan), recognized as a medium-intensity conditioning strategy (89). Moreover, low-intensity, or non-myeloablative, regimens typically incorporate CY and ATG (90), with ongoing discussions about the optimal conditioning methodology. Subsequent to conditioning, cryopreserved HSCs are thawed and reintroduced to the patient’s bloodstream. The application of ATG at this stage aims to eliminate any residual autoreactive T cells that might have survived the HDIT. This phase marks the beginning of the patient’s recovery, characterized by a critically reduced hematopoietic cell count, necessitating prophylactic antiviral and antibacterial measures (91).

#### 4.1.2 Mechanisms of HSCs treatment

HSCs is increasingly recognized for its potential in managing MS, yet the precise immunological pathways contributing to its therapeutic impact are not fully understood. Currently, two main hypotheses are proposed to explain its efficacy. The first and more widely accepted hypothesis suggests that HSCT’s success in MS treatment stems from its ability to ‘reset’ the immune system. By introducing healthy HSCs, HSCT eliminates the dysfunctional immune cells, paving the way for the formation of a renewed immune system. This renewal process re-establishes immune tolerance and regulatory functions, effectively curbing the patient’s autoimmune activity (28, 92). The reconstitution of immune tolerance is thought to be facilitated by regulatory T cells, which play a key role in suppressing Th17-mediated inflammatory and autoimmune responses, thus reducing the emergence of autoreactive T cells. Following immune system reconstitution, there is a notable decline in pathogenic CD4+ Th17 lymphocyte levels. Peripheral blood CD8+ T cell counts may normalize within three months post-aHSCT, with B lymphocyte levels returning to baseline after six months (93). In patients with MS undergoing aHSCT, a significant diminution in Th17 and Th1 cell activity is observed. Research employing microarray DNA chip technology has revealed significant changes in the gene expression profiles of peripheral CD4+ and CD8+ T cell subsets after aHSCT (94). This may be due to the depletion of autoreactive cells by HSCT, followed by the replacement and replenishment of adaptive immune cells. Harris et al. evaluated the T-cell repertoire in paired cerebrospinal fluid (CSF) and peripheral blood CD4+ and CD8+ T cells from active RRMS patients undergoing aHSCT. They found that after aHSCT, over 90% of the pre-existing CSF repertoire was removed, and replaced by clonotypes generated from transplanted autologous stem cells (95). This underscores the recalibration of the immune system’s pro-inflammatory and immunoregulatory components following HSCT, facilitating the reestablishment and functional repair of the immune system in MS.

Another explanation for the therapeutic effect of HSCT may involve the prolonged T-cell depletion of the conditioning regimen, leading to persistent immune quiescence, thereby eliminating significant autoimmune activity (96). However, due to severe T-cell depletion, the incidence of infections and the risk of graft-versus-host disease (GvHD) with HSCT increase. Various conditioning regimens, including the CliniMACs system and donor lymphocyte infusions (DLIs), have been proposed to prevent the occurrence of infections and GvHD (97). Indeed, tissue-resident memory T cells, which are antigen-experienced T cells permanently residing in barrier tissues, are unlikely to be completely depleted (98). However, whether complete tissue immune cell depletion is necessary or whether the maintenance of tissue immune cells can protect patients from post-transplant complications such as infections remains a subject of profound discussion and research evidence. Future studies may elucidate the prolonged depletion of tissue immune cells post-transplantation and whether this affects clinical outcomes.

#### 4.1.3 Clinical evidence for HSCs

A series of clinical studies have been conducted to explore the efficacy of HSCs in treating MS. Fassas et al. pioneered this approach in 1997, applying peripheral blood stem cell transplantation to patients with progressive MS. Post-transplantation, a significant reduction in CD4+ cell counts was observed across the cohort, while CD8+ cells increased by approximately 50% at the 3-month mark, gradually decreasing thereafter but remaining above baseline CD4+ levels. Neurological function, assessed via the Scripps Neurological Rating Scale (SNRS), improved, suggesting that peripheral blood HSCT is relatively safe and does not exacerbate the disease (84). This discovery marks the beginning of preliminary clinical research on HSC therapy for MS, providing a direction for subsequent more in-depth and extensive studies.

Recent studies have rigorously evaluated the efficacy of aHSCT in a cohort of 507 MS patients. This comprehensive single-center study included 414 patients with RRMS and 93 with SPMS, all treated with non-myeloablative aHSCT, and reported an impressive 5-year survival rate of 98.8% (99). Moreover, a multicenter retrospective analysis revealed that MS patients treated with aHSCT experienced no clinical relapses or further disability progression. Six months post-transplantation, MRI evaluations confirmed the stability of the treatment’s effects. Remarkably, 95% of patients showed improvements in Kurtzke Expanded Disability Status Scale (EDSS) scores, signifying enhanced neurological functions (100). The aHSCT is considered as a potential treatment for MS, becoming widely used in clinical practice and showing obvious advantages. In a single-center cohort study, Vivien Häußler et al. compared the outcomes of disease activity in patients with MS undergoing aHSCT or treatment with alemtuzumab. The study results revealed significant improvements in EDSS and cognitive function in patients receiving aHSCT treatment compared to those receiving alemtuzumab therapy, and the aHSCT group maintained longer periods of no evidence of disease activity (NEDA). These findings suggest that aHSCT may be more effective than alemtuzumab in improving overall disability and cognitive abilities in MS (101). Another investigation compared the long-term disability progression between aHSCT recipients and patients receiving standard DMTs in a cohort of active SPMS patients. This study involved 79 individuals undergoing aHSCT and 1975 receiving DMTs such as interferon-beta, azathioprine, and others. Results indicated a significant delay in the time to first confirm disability progression among aHSCT recipients, suggesting that aHSCT may slow disability advancement and enhance the probability of functional improvement in active SPMS patients compared to conventional immunotherapy (102). These studies highlight the efficacy of HSCT in the treatment of MS and its advantages over other treatment modalities, providing strong evidence for the clinical application of HSCs in MS practice.

#### 4.1.4 Clinical safety of HSCs treatment

Current research on the safety profile of HSCT acknowledges its therapeutic successes but also highlights inherent risks, including infections and suppression of hematopoietic functions. Initial adverse effects typically involve fever and viral infections, with more delayed risks possibly encompassing autoimmune thyroiditis (99, 103). Silfverberg et al. conducted a retrospective analysis of 174 RRMS patients undergoing aHSCT, revealing that among 149 baseline disability patients, 54% showed improvement and 37% remained stable. Additionally, febrile neutropenia was the most common adverse event, with no treatment-related deaths reported (104). In a retrospective single-center observational study of all MS patients undergoing HSCT, 43% of patients showed sustained improvement in EDSS scores, 17% were diagnosed with autoimmune thyroid disease post-surgery, and 43% of women experienced amenorrhea and ovarian failure without any reported fatalities (105). Despite acceptable adverse events, the use of HSCT for MS treatment yields a higher benefit-to-risk ratio. Contemporary studies increasingly prioritize evaluating the safety and enduring impacts of HSCT, with a particular focus on reducing adverse reactions through refined dosing and drug regimens. Recent analyses have scrutinized the risk of secondary autoimmune events after various preconditioning protocols. Specifically, the incidence of complications was higher in myeloablative busulfan-based and non-myeloablative protocols, recorded at 18 and 7.7%, respectively. Conversely, the BEAM-aHSCT protocol exhibited a substantially reduced risk, at less than 1% (106). Further examination of BEAM-ATG and Cy-ATG approaches revealed similar risks, with a notable increase in secondary autoimmune thyroiditis among a cohort of 139 aHSCT recipients. The latest cohort study observed an increase in secondary autoimmunity rates post-aHSCT from 6% (107) to 17% (105), underscoring the need for vigilant monitoring and management of these risks. These findings are crucial for shaping future MS treatment protocols.

It is noteworthy that the risk of immune rejection poses a significant barrier to stem cell therapy, particularly for MS patients with hyperactive immune responses. Post-transplant GvHD remains a major cause of treatment failure and increased mortality rates in HSCT (108). Predicting the occurrence of immune rejection can be facilitated by pre-transplantation assessment of donor-derived hematopoietic stem cell expression markers, such as IL12 and IFNγ (109), thereby enabling conditioning or mobilization of patients to enhance the effectiveness and safety of HSCT. However, further research is needed to explore the incidence and risk of immune rejection after HSCT in MS, as well as effective measures to prevent its occurrence.

### 4.2 Application of mesenchymal stem cells in MS

The use of MSCs therapy is one of the rapidly developing branches of regenerative medicine. The simplicity of obtaining MSCs, along with their low immunogenicity and immunomodulatory capabilities, means they can be transplanted into autologous and allogeneic systems (110). Utilizing MSCs for stem cell therapy has shown promising prospects in the treatment of MS.

#### 4.2.1 Mechanisms of MSCs treatment and preclinical evidence

Although the exact mechanisms underlying the therapeutic benefits conferred by MSCs remain to be fully elucidated, MSCs exhibit potential therapeutic efficacy due to their neuroregenerative, neuroprotective, and immunomodulatory properties (111). BM-MSCs have been shown to possess neuroprotective and remyelinating abilities under certain experimental conditions. Studies have revealed that BM-MSCs can migrate to damaged CNS in chronic and relapsing–remitting EAE mouse models, reducing injury severity, and increasing oligodendrocyte lineage cell presence in the lesion area, thus promoting functional recovery. Additionally, BM-MSCs also influence the host’s immune response, characterized by a decrease in inflammatory T cells, including interferon-γ-producing Th1 cells and interleukin-17-producing Th17 cells, and an increase in anti-inflammatory T cells, such as interleukin-4-producing Th2 cells (112). Moreover, transplanted MSCs can inhibit demyelination and stimulate remyelination, with newly formed myelin sheaths observed around axons in the corpus callosum and spinal cord during acute EAE (113). Transplantation of MSCs derived from full-term human placenta (PDMSCs) reduces brain inflammation and neurodegeneration in EAE rats, significantly improving disease course, and significantly expressing human brain-derived neurotrophic factor (BDNF), nerve growth factor (NGF), and neurotrophin-3 (NTF3) (114). The phenomenon of gaining direct neural support through the expression of neurotrophic factors post-MSCs implantation has garnered widespread attention.

Recent researchers have termed the mechanism by which MSCs exert neuroprotective effects through the secretion of bioactive substances as MSC-secretome (115). The MSC-secretome comprises cytokines, growth factors, microRNAs, etc., encapsulated in EVs such as exosomes (116). A study by Bai et al. demonstrated that hepatocyte growth factor (HGF) secreted by MSCs could improve memory defense and functional recovery in EAE mice, promoting the development of oligodendrocytes and neurons, and playing a crucial role in remyelination (117). Gratpain et al. found that SCAP can reduce the expression of pro-inflammatory markers in microglial cells by modifying the miRNA content of their secreted EVs during their study on the impact of SCAP secretome on microglial cells (118). Furthermore, hPDLSCs from RRMS patients can modulate the expression of inflammatory cytokines (TNF-α, IL-10), neuroprotective markers (Nestin, NFL70, NGF, GAP43), and apoptotic markers (Bax, Bcl-2, p21) in mouse motoneurons. Importantly, the EV from hPDLSC significantly express anti-inflammatory cytokines IL-10 and TGF-β. These findings suggest that hPDLSCs from RRMS patients exhibit immunosuppressive effects on inflamed motor neurons (119). A recent meta-analysis summarized the therapeutic effects of MSC-EVs in rodent models of MS, showing that MS animals benefited from MSC-EVs treatment, significantly improving clinical symptoms and delaying disease progression (120). Although preclinical studies suggest that MSC-EVs can improve MS, several questions remain unanswered regarding the timing, route, and dosage of MSC-EVs administration. Ahmadvand Koohsari et al. found that intravenous injection of human umbilical cord MSC-derived extracellular vesicles (hUCSC-EVs) reduced pro-inflammatory cytokines such as IL-17a, TNF-α, and IFN-γ, and leukocyte infiltration while increasing anti-inflammatory cytokines IL-4 and IL-10, thereby alleviating EAE symptoms (5).

MSCs possess potent immunomodulatory properties and can promote allograft tolerance (121). Zhang et al. found that MSCs can reduce neuroinflammation in EAE mice by increasing the M2 phenotype of microglia and decreasing their M1 phenotype and associated cytokines (122). Transplantation of BM-MSCs improves the immune mechanisms of EAE, including inhibiting T cell proliferation and activation, reducing the production of inflammatory cytokines, and modulating macrophage responses, particularly macrophage polarization, thereby preventing the onset of EAE (123). These findings broaden our understanding of MSCs transplantation in regulating T cell and macrophage immune responses. Additionally, MSCs can inhibit the proliferation of pro-inflammatory cell subsets (Th17 and Th1) and reduce the Th1/Th2 ratio of helper T cell subsets, promoting anti-inflammatory features by activating Treg cells (124). MSC-induced Treg cell activation is achieved by increasing the demethylation of Treg-specific demethylated regions (TSDR) and upregulating the expression of the Runx composite genes in TSDR, including Foxp3 (Runx1, Runx3, and CBFB) (125). MSC-induced Tregs are believed to be mediated by the secretion of PGE2, TGFβ1, IL10, and soluble human leukocyte antigen-G (sHLA-G) (126). The balance between Treg cells and T cells determines the effectiveness of immunotherapy, emphasizing the importance of MSCs as tools for regulating autoimmunity and treating MS.

#### 4.2.2 Clinical evidence for MSCs

Insights from EAE treatments have propelled MSCs therapy into clinical trials. In a pioneering clinical study, autologous MSCs therapy was administered intrathecally to 10 PMS patients, with monitoring periods ranging from 13 to 26 months. Autologous MSCs therapy led to a modest improvement in clinical symptoms (127). Small-scale studies have provided supporting data on the safety and potential efficacy of single-dose MSCs therapy. Bonab et al. reported favorable clinical or MRI outcomes in 15 out of 25 patients, suggesting MSCs therapy as a viable option for MS patients unresponsive to standard treatments (128). The International Mesenchymal Stem Cell Transplantation Study Group (IMSCTSG) initiated a Phase I/II trial to evaluate the efficacy of autologous MSCs therapy in MS patients. Participants were divided into two cohorts, one receiving an intravenous infusion of autologous bone marrow-derived MSCs and the other a matching placebo. The trial demonstrated that MS patients receiving MSCs therapy showed a reduction in the number of new lesions and a significant decrease in lesion volume within six months of treatment (129). Neurofilament light chain (NF-L) and the chemokine receptor CXCL13 are important biomarkers for assessing MS. Karussis et al. conducted a double-blind randomized phase II clinical trial to evaluate the levels of neurofilament light chain (NF-L) and CXCL13 in the CSF of patients with progressive MS following treatment with MSCs. The results revealed a decrease in NF-L and CXCL13 levels in the CSF of patients 6 months after MSCs transplantation. The reduction in NF-L levels was significant, but the decrease in CXCL13 levels did not reach statistical significance (130). Therefore, MSCs transplantation may have neuroprotective effects on patients with MS.

Building upon the initial promising outcomes of MSCs therapy in the treatment of MS, researchers focus on exploring the diverse administration methods of MSCs for MS management. In a Phase II study, employing a randomized, placebo-controlled framework, Llufriu et al. explored changes in clinical assessments, brain MRI findings following intravenous MSCs therapy. The results confirmed the safety of the procedure and suggested a reduction in inflammatory MRI markers six months after treatment (131), providing a foundation for further investigation into MSCs therapy’s therapeutic potential in MS management. Mohyeddin Bonab et al. found that intrathecal injection of MSCs could ameliorate the condition of MS patients. However intrathecal delivery of MSCs did not alter cytokine profiles but resulted in an uptick in regulatory T-cell counts and a reduction in lymphocyte proliferation rates (132). Petrou and Kassis et al. investigated the safety and clinical efficacy of MSCs transplantation in patients with RRMS and progressive MS, as well as evaluating the optimal route of administration. Following a 14-month study involving 48 patients with MS, they found no treatment-related serious safety concerns among those who received MSC transplantation. Compared to the placebo group, recipients of bone marrow mesenchymal stem cell transplantation experienced reduced disease relapse rates and demonstrated more beneficial therapeutic effects on imaging and cognitive tests. Additionally, regarding the route of administration, intrathecal delivery outperformed intravenous administration across multiple disease parameters (133). Although intravenous and intrathecal administration of MSCs has shown distinct therapeutic advantages, the optimal route of administration for MSCs therapy in MS has not been sufficiently validated. Furthermore, active exploration of effective management strategies for MSCs should be pursued to expand their application in MS treatment.

Research on the therapeutic use of MSCs for MS includes both autologous and allogeneic sources. In MS clinical trials, there is a predilection for allogeneic MSCs harvested from fetal tissues such as placenta, amniotic epithelial cells, umbilical cord, umbilical cord matrix, and Wharton’s jelly (134). A Phase II trial involving repeated intravenous infusions of UC-MSCs reported a decrease in clinical symptoms and relapse frequency in MS patients, with serum analyses indicating a shift from a Th1 (pro-inflammatory) to a Th2 (anti-inflammatory) immune response (135). Additionally, a combined Phase I/II study focusing on SPMS patients observed a decline in relapse rates and/or lesion intensity, along with clinical score improvements following UC-MSCs therapy (8). These findings underscore the importance of further exploring the therapeutic potential of MSCs from different sources in the treatment of MS.

#### 4.2.3 Clinical safety of MSCs treatment

Regarding treatment safety, earlier studies affirm the general safety of MSC transplantation in MS patients, with clinical data showing minimal adverse effects. However, some reports highlight potential mild side effects, including fever and headaches. A multicenter placebo-controlled study corroborated that intravenous MSCs administration does not influence the number of lesions (136). A recent meta-analysis reviewed adverse events in various disease populations following MSCs administration, revealing that the therapy is safe and closely associated only with mild adverse reactions such as short-term fever, local adverse events at the administration site, constipation, fatigue, and insomnia (137). In a study conducted by Danbour et al., within a Phase I/IIa prospective clinical trial framework, the safety and practicability of using BM-MSCs for treating MS were assessed. The findings revealed that the patients exhibited good tolerance to the treatment regimen, with improvement trends noted in all other assessments, and no significant adverse events occurred (36). It is noteworthy that in a randomized, double-blind phase II clinical trial conducted by Ucelli et al., the viewpoint opposing the use of BM-MSCs for treating active MS was presented. This study conducted at 15 sites in 9 countries, and aimed to evaluate the safety, tolerability, and efficacy of autologous BM-MSCs. They reported 213 adverse events, with the most common being infections. Furthermore, no serious adverse events were observed in the BM-MSCs transplantation group compared to the placebo group. Their study suggest that BM-MSCs therapy is safe and well-tolerated. However, at the 24-week mark, MSCs transplantation did not improve acute inflammation in MS patients as assessed by gadolinium-enhancing lesions and MRI surrogate marker. Therefore, further research is warranted to elucidate the effects of BM-MSCs on tissue repair of MS (138). Recent research has pivoted towards autologous mesenchymal stem cell-derived neural progenitor cells (MSC-NP) as an alternative to BM-MSCs, aiming to reduce ectopic differentiation risks in the CNS post-transplantation. Initial results from a Phase I trial indicate that autologous MSC-NP transplantation is not only safe but also well-tolerated. Further investigations reinforce the positive safety and efficacy profile of MSC-NP transplantation, offering substantial evidence of its viability as an alternate therapeutic option (139).

### 4.3 Application of neural stem cells in MS

#### 4.3.1 Mechanisms of NSCs treatment

The beneficial effects of NSCs are attributed to a variety of mechanisms, such as cellular replacement, immunomodulation, support of endogenous repair through nutritional factors, and enhancement of progenitor cell differentiation (140). NSCs possess the ability to differentiate into key neural cell types, including neurons, astrocytes, and oligodendrocytes (42). In the EAE model, NSCs are known to become activated and migrate towards areas of inflammation and demyelination within the central nervous system, where they can differentiate into oligodendrocytes, offering therapeutic promise (141). Brown et al. found that NSCs homed to the central nervous system and potentially differentiated into neural derivatives, promoting neurogenesis and myelination through modulation of the BDNF and FGF signaling pathways. Additionally, NSCs were implicated in regulating Treg and Th17 cell levels in EAE mice, inducing anti-inflammatory responses and reducing immune infiltration (45).

Some research suggests that NSCs exhibit immunomodulatory effects both locally and systemically, leading to decreased perivascular cell infiltration, lower CD3+ cell counts, and reduced expression of ICAM-1 and LFA-1 (142). Additionally, an increase in Treg cell populations has been noted in both the brain and spinal cord (143), highlighting another dimension of NSCs’ therapeutic potential. Notably, intravenous NSC transplantation has been shown to reduce the presence of CD3+ T cells and Mac3+ macrophages within the spinal cord, indicating a direct immunomodulatory effect (144). NSCs further exhibit the capacity to inhibit T-cell proliferation and cytokine production. Supporting data points to the role of soluble mediators in NSCs’ immunosuppressive functionality, with the leukemia inhibitory factor (LIF) emerging as a key factor in this immunomodulatory mechanism (145).

Different studies have indicated that neural stem cells can regulate central nervous system development and function by producing neurotrophic factors such as NGF, vascular endothelial growth factor (VEGF) (146), neurotrophin-3 (NT3) (147), and insulin-like growth factor (IGF)-1 (148). The “secretome” of NSCs and its correlation with disease improvement in animal models of neurodegenerative diseases have recently gained significant attention. Lee et al. found that NSCs cultured *in vitro* could produce neurotrophic factors, including BDNF, NGF, and VEGF. Neural stem cells migrate extensively from the injection site and differentiate into neurons and glial cells. Moreover, spatial memory impairment in treated mice showed some improvement (146). However, the correlation between the secretome of neural stem cells and MS has been poorly studied, requiring further research to elucidate the complex molecular signaling regulated by the NSCs secretome.

#### 4.3.2 Preclinical evidence for NSCs

The therapeutic potential of NSCs in MS has been demonstrated through a range of preclinical studies. In EAE, the administration of NSCs, whether via intravenous infusion or direct transplantation into the lateral ventricles from the SVZ, has led to significant functional improvements across various disease stages, including pre-onset, onset, and peak phases (149). Additionally, during the chronic phase of EAE, intravenous NSC delivery has been shown to enhance functional recovery by inducing apoptosis in pro-inflammatory T cells and reducing the infiltration of inflammatory immune cells (150). Peruzzotti-Jametti et al. have shown that NSCs can alter the pro-inflammatory behavior of mononuclear phagocytes (MPs) by secreting anti-inflammatory prostaglandin E2 (PGE2) and sequestering the extracellular immunometabolite succinate. This interaction prompts a metabolic shift in MPs, facilitated by direct contact between NSCs and MPs in the meninges’ perivascular areas. This metabolic reprogramming contributes to the reduction of chronic neuroinflammation in EAE mice, thereby supporting functional recuperation (151). In EAE models using non-human primates, hNSCs have demonstrated therapeutic efficacy, markedly reducing disease severity, improving functional outcomes, and extending survival (152). Following xenogeneic transplantation, hNSCs were found to localize around blood vessels in inflamed areas of the CNS, effectively inhibiting T cell proliferation and dendritic cell maturation (145). While initial expectations centered on hNSCs differentiating into neural cells and integrating into the damaged CNS, recent preclinical findings suggest that their therapeutic effects are primarily mediated through immunomodulation, enhancement of neuroprotection, and restoration of internal homeostasis (153). In conclusion, while preclinical studies have demonstrated the therapeutic potential of NSCs in MS, primarily through immunomodulation and neuroprotection, their clinical translation faces limitations. The forthcoming discussion will explore clinical evidence regarding NSCs therapy in MS treatment.

#### 4.3.3 Clinical evidence and safety for NSCs

Clinical studies on NSCs therapy for MS have not yet reached a stage of widespread enthusiasm. Current clinical research on NPCs largely focuses on NPCs derived from mesenchymal stem cells (MSC-NP). A Phase I clinical trial evaluated the safety and tolerability of autologous MSC-NP therapy in 20 progressive MS patients. This trial confirmed the therapy’s safety, with participants showing good tolerability and no serious adverse effects reported. Mild side effects, such as temporary fever and slight headaches, were observed but typically subsided within 24 h. Furthermore, after receiving MSC-NP therapy, 70% of the participants reported enhanced muscle strength, and 50% noted improvements in bladder control (139). A comprehensive evaluation was conducted two years post-treatment to ascertain the long-term safety and effectiveness of repeated intrathecal administrations of autologous MSC-NP in progressive MS patients. Among the 20 participants who underwent MSC-NP therapy, 18 reported no long-lasting adverse effects. Notably, seven patients exhibited sustained improvements in their EDSS scores. Further analysis of cerebrospinal fluid biomarkers showed a reduction in CCL2 levels and an increase in IL-8, hepatocyte growth factor, and CXCL12 after the therapy. These changes in biomarkers might reflect the unique immunomodulatory and trophic actions of MSC-NP therapy in MS management (154). A study assessing the feasibility, safety, and tolerability of the transplantation of allogeneic human neural stem/progenitor cells (hNSCs) in SPMS involved a one-year follow-up of 15 patients. The results demonstrated that patients receiving intraventricular hNSC injections, alongside immunosuppressive treatment, experienced no treatment-related deaths or serious adverse events (155). A research team conducted a Phase I clinical trial characterized by a single-dose administration in a non-randomized, open-label format, involving the transplantation of fetal neural stem/precursor cells. These cells, derived from the cerebral tissue of aborted fetuses, were transplanted into the spinal cords of patients with progressive MS. The trial reported positive shifts in disease biomarkers among participants, without any adverse effects linked to the treatment. Three months following the procedure, significant increases in neurotrophic factors and anti-inflammatory agents were observed in the patient’s cerebrospinal fluid, indicating the potential neuroprotective effects of the transplanted stem cells (156).

These compelling results affirm the sustained safety and therapeutic potential of NSCs in treating MS. However, the limited availability of NSCs poses challenges for widespread clinical application, prompting the exploration of more accessible NSCs sources. Consequently, there is heightened interest in the preclinical study of ESCs and iPSCs as viable alternatives.

### 4.4 Application of embryonic stem cells in MS

#### 4.4.1 Mechanisms of ESCs treatment and preclinical evidence

Due to ethical concerns surrounding hESCs research, the application of hESCs transplantation in autoimmune diseases remains a topic of debate. While the specific mechanisms by which ESCs therapy exerts its effects in MS remain to be fully elucidated, several hypotheses have been posited. These include hESCs’ differentiation into neural cell types such as neurons, astrocytes, and oligodendrocytes, their role in reducing apoptosis, modulating neurotrophic factor release, and mitigating inflammatory responses (157). In primate models of EAE, the intrathecal delivery of extramedullary mesenchymal stem cells (EMSCs) derived from hESCs led to notable improvements in clinical outcomes, reduction of brain pathology, and protection against neuronal demyelination. In contrast, the control group exhibited progressive enlargement of MRI-detected brain lesions. EMSCs demonstrated the ability to differentiate into neural cell types in the CNS, alongside an increase in the expression of genes related to neuronal markers, neurotrophic factors, and myelination processes. These results suggest that direct intrathecal administration of EMSCs can decelerate disease progression, underscoring the potential for clinical application of embryonic stem cell therapies (158). Furthermore, transplantation of neural progenitor cells derived from hESCs has been shown to alleviate clinical symptoms in EAE mice. Although transplanted neural progenitor cells were observed in the mouse brain, remyelination and the generation of mature oligodendrocytes were not observed. Clinical improvements may be attributed to immunosuppression and neuroprotective mechanisms (63). Recent studies have reported the derivation of pluripotent stem cells from mouse fibroblasts through reprogramming with specific transcription factors (65), thereby enhancing the prospect of utilizing potential autologous cell sources from hESC derivatives and avoiding ethical concerns associated with human embryo usage. Reports have demonstrated the ability of mESCs to generate NSCs *in vitro* using developmental cues (159), including region-specific neuronal subtypes such as dopaminergic neurons and motor neurons (160). Despite interspecies differences, insights into the neurogenic potential of mESC provide information and a platform for ESC research.

#### 4.4.2 Clinical evidence for ESCs

One case highlighted a 34-year-old female MS patient who received hESC transplantation, with subsequent diffusion tensor imaging revealing marginal decreases in lesion sizes near the bilateral ventricles and adjacent to the right occipital lobe’s white matter. In another investigation, two patients with concurrent diagnoses of MS and Lyme Disease (LD) exhibited significant improvements in functional abilities, endurance, cognitive function, and muscle strength following hESC therapy, as assessed by diffusion tensor imaging and single-photon emission computed tomography. These outcomes suggest the efficacy and safety of hESC therapy for patients with MS and LD (161). However, the current body of evidence remains limited, underscoring the need for comprehensive clinical trials to confirm the long-term efficacy and safety of hESC therapy in MS. Research in this area continues to face various technical and ethical challenges, including issues related to the sourcing of ESCs and controlling their differentiation. Additionally, the regulatory environment surrounding the sourcing and application of ESCs, involving ethical debates and the necessity of donor consent, adds further complexity to the widespread use of ESCs. Research in this field must navigate within established legal and ethical frameworks.

### 4.5 Application of induced pluripotent stem cells in MS

iPSCs are a dynamic category of cells that can be reprogrammed from various somatic tissues, possessing the capability to differentiate into oligodendrocyte precursor cells (OPCs). This characteristic positions iPSCs as a promising candidate for autologous cell therapy approaches (162). Current preclinical research is actively exploring the therapeutic potential of iPSCs, with preliminary findings indicating that iPSCs-derived OPC can mitigate both clinical symptoms and pathological changes in EAE, largely through neuroprotective mechanisms rather than direct remyelination (163). The administration of iPSC-derived neural progenitor cells (iPSC-NPCs) into EAE models has demonstrated significant benefits, including reduced infiltration of inflammatory cells, decreased spinal cord demyelination, and lessened axonal damage. The therapeutic impact of iPSC-NPCs in these models is attributed to their secretion of neuroprotective factors like LIF, enhancing the survival and maturation of oligodendrocytes (164). Another study highlighted the significant decrease in T-cell infiltration and attenuation of white matter damage following iPSC-NSCs transplantation in EAE. Treatment with iPSC-NSCs also resulted in notable reductions in disease symptom scores and enhancements in motor skills, affirming the potential of iPSC-NSCs as a therapeutic option for MS (165).

Moreover, astrocytes have been shown to play multiple roles in the injury and repair processes of MS (166). Kerkering et al. differentiated iPSCs derived from BMS patients into NSCs, further differentiating them into BMS patient-specific neurons and astrocytes. They found that iPSC-derived astrocytes exerted a protective effect against TNF-α/IL-17-induced neuronal pathology. This neuroprotective effect was mediated through the JAK/STAT signaling pathway. Activation of the JAK/STAT pathway induced the production of soluble mediators such as LIF, BDNF, and TGF-β1, which exerted neuroprotective effects. Additionally, iPSC-induced astrocytes from BMS patients stabilized the TNF-α-induced NF-κB signaling pathway, thereby protecting cells from inflammatory neuronal damage (167).

With the successful reprogramming of somatic cells into iPSCs, innovative strategies for direct neural lineage conversion have been developed. The application of neural lineage-specific transcription factors (TFs) facilitates the creation of induced neural stem cells (iNSCs). When transplanted into models of EAE, iNSCs have shown the capacity to differentiate into oligodendrocytes and integrate into disrupted myelin structures in the brain (168). Yun et al. also manipulated human somatic cell reprogramming into OPCs by combining OCT4 with small molecules. They transplanted the generated iOPCs into the brains of EAE mice and observed that OPCs or iOPCs could integrate into the host nervous system by day 100 post-transplantation. They differentiated into mature oligodendrocytes, significantly ameliorating disease symptoms to levels comparable to normal mice and showing no tumorigenic effects. Moreover, transmission electron microscopy (TEM) of the brains and spinal cords of mice in the iOPC and OPC transplantation groups revealed abundant compact myelin when compared to the PBS group, indicating that transplanted iOPCs and OPCs could promote axonal remyelination (169).

Notably, there is concern that donor cells may retain epigenetic memory post-reprogramming, raising the possibility of immune rejection after transplantation. Additionally, the lengthy process of generating iPSC-derived NSCs carries the risk of introducing genetic instabilities, increasing tumorigenesis potential (163). Therefore, while iPSCs and their neural derivatives show promise in preclinical studies, significant obstacles remain in their path to clinical use. To enhance the efficacy of neural repair, it is essential to precisely guide stem cell differentiation into specific neuronal cell types, a process involving intricate control mechanisms of gene expression and signaling pathways (170). A thorough understanding of the factors regulating differentiation is crucial to ensure reliable guidance of stem cells towards functional neuronal cells. Future research is necessary to explore the ability of transplanted stem cells to maintain stable differentiation *in vivo* and prevent unwanted cell proliferation or differentiation.

## 5 Conclusion

In summary, traditional therapies for multiple sclerosis (MS) are limited by side effects and varying efficacy, contrasting sharply with the overall prospects of stem cell therapies aimed at neuroregeneration, neuroprotection, and immunomodulation. Various types of stem cells, including HSCs, MSCs, NSCs, ESCs, and iPSCs, have demonstrated significant therapeutic potential for MS in preclinical and clinical studies. Simultaneously, exploration of the effectiveness and safety of SCs in treating MS is gradually advancing. Despite the initial success of these stem cells in MS treatment, significant challenges persist, including regulation of neural induction differentiation, immune rejection, and ethical oversight. Future research needs to not only refine these areas but also explore combination therapies to enhance treatment outcomes. The development of future stem cell therapies could significantly alter the landscape of MS treatment. The path forward will require balanced collaborative efforts to translate promising preclinical findings into safe, effective clinical applications, offering new hope for patients with MS.

## Author contributions

LW: Conceptualization, Resources, Supervision, Writing – review & editing. JL: Conceptualization, Resources, Supervision, Writing – review & editing. TL: Conceptualization, Resources, Supervision, Writing – review & editing. DZ: Data curation, Formal analysis, Writing – original draft. HX: Data curation, Formal analysis, Writing – original draft. ZK: Investigation, Methodology, Writing – review & editing. FP: Investigation, Methodology, Writing – review & editing. JW: Investigation, Methodology, Writing – review & editing.
